# Atypical cannabinoid ligands O-1602 and O-1918 administered chronically in diet-induced obesity

**DOI:** 10.1530/EC-18-0535

**Published:** 2019-02-01

**Authors:** Anna C Simcocks, Kayte A Jenkin, Lannie O’Keefe, Chrishan S Samuel, Michael L Mathai, Andrew J McAinch, Deanne H Hryciw

**Affiliations:** 1 Institute for Health and Sport, Victoria University, St Albans campus, Melbourne, Victoria, Australia; 2 School of Science and Health, Western Sydney University, Campbelltown, New South Wales, Australia; 3 Cardiovascular Disease Program, Biomedicine Discovery Institute and Department of Pharmacology, Monash University, Clayton, Victoria, Australia; 4 The Florey Institute of Neuroscience and Mental Health, Parkville, Melbourne, Victoria, Australia; 5 Australian Institute for Musculoskeletal Science (AIMSS), College of Health and Biomedicine, Victoria University, Melbourne, Victoria, Australia; 6 School of Environment and Sciences, Griffith University, Nathan, Queensland, Australia

**Keywords:** cannabinoid, GPR55, GPR18, high-fat diet, O-1602, O-1918, obesity

## Abstract

Atypical cannabinoid compounds O-1602 and O-1918 are ligands for the putative cannabinoid receptors G protein-coupled receptor 55 and G protein-coupled receptor 18. The role of O-1602 and O-1918 in attenuating obesity and obesity-related pathologies is unknown. Therefore, we aimed to determine the role that either compound had on body weight and body composition, renal and hepatic function in diet-induced obesity. Male Sprague–Dawley rats were fed a high-fat diet (40% digestible energy from lipids) or a standard chow diet for 10 weeks. In a separate cohort, male Sprague–Dawley rats were fed a high-fat diet for 9 weeks and then injected daily with 5 mg/kg O-1602, 1 mg/kg O-1918 or vehicle (0.9% saline/0.75% Tween 80) for a further 6 weeks. Our data demonstrated that high-fat feeding upregulates whole kidney G protein receptor 55 expression. In diet-induced obesity, we also demonstrated O-1602 reduces body weight, body fat and improves albuminuria. Despite this, treatment with O-1602 resulted in gross morphological changes in the liver and kidney. Treatment with O-1918 improved albuminuria, but did not alter body weight or fat composition. In addition, treatment with O-1918 also upregulated circulation of pro-inflammatory cytokines including IL-1α, IL-2, IL-17α, IL-18 and RANTES as well as plasma AST. Thus O-1602 and O-1918 appear not to be suitable treatments for obesity and related comorbidities, due to their effects on organ morphology and pro-inflammatory signaling in obesity.

## Introduction

The prevalence of obesity is increasing worldwide and is an established risk factor for a number of comorbidities including diabetes, hypertension, chronic kidney disease and fatty liver disease (1, 2). The significant social and financial burden associated with obesity has warranted the investigation of therapeutic targets to reduce the pathophysiological changes observed in obesity. One pharmacological target currently being investigated for obesity and associated comorbidities is the endocannabinoid system (3). In obesity, the endogenous endocannabinoid anandamide (AEA) promotes appetite and reduces energy expenditure through the activation of cannabinoid receptor 1 (CB_1_) (4). AEA and tetrahydrocannabinol (THC) binds with a similar affinity to cannabinoid receptors CB_1_ and CB_2_ (5). Research into the dysregulated endocannabinoid system in obesity has been investigated for more than a decade (6, 7, 8).

One atypical cannabinoid derived from *Cannabis sativa* is cannabidiol (CBD). This compound has a number of physiological functions such as reducing inflammation and oxidative stress (9). A synthetic isomer of CBD is abnormal cannabidiol (Abn-CBD) (10). Both Abn-CBD and CBD have limited binding capacities to CB_1_ and CB_2_. Consequently, these compounds do not induce the psychotropic effects induced by THC (4). CBD has long lasting effects (up to 80 days) and has been used for the treatment of inflammatory pain and multiple sclerosis (MS) in mouse models (11). In humans CBD treatment safely and effectively reduces symptoms of pain and spasticity in MS patients (12). Additionally, both CBD and Abn-CBD mediate a potentially protective role in diabetes (13, 14). Although, in humans with T2DM, CBD does not have the same protective effects that are observed in diabetic animal and cell culture models (15, 16, 17). CBD also promotes a browning phenotype, lipolysis, thermogenesis and reduces lipogenesis in 3T3-L1 adipocytes (18).

CBD and Abn-CBD have an affinity to putative cannabinoid receptors G protein-coupled receptor 55 (GPR55) and G protein-coupled receptor 18 (GPR18) (9, 19, 20). O-1918, a synthetic compound similar to CBD, is a putative antagonist for GPR55 and an antagonist for GPR18 (20) or a biased agonist for GPR18 (21). Limited research has been conducted examining the potential therapeutic use of O-1918 in disease. *In vitro*, O-1918 may be useful in promoting wound healing and bone regeneration, as treatment with O-1918 in mesenchymal stem cells increases migration in a concentration-dependent manner via the p44/42 MAPK pathway (22). O-1918 may also mediate cardiovascular hemodynamics, as this compound can inhibit the hypotensive effects of Abn-CBD and AEA (23).

Conversely, O-1602 is a synthetic analog of Abn-CBD and is also a potent agonist for GPR55 (24) and a biased agonist for GPR18 (21). Using animal models, O-1602 mediates a number of physiological effects including a reduction in pain (25) and inflammation (26), an inhibition in tumor growth (27), an inhibition of osteoclast formation *in vitro* (28), an inhibition of neutrophil migration (29) as well as regulating gastrointestinal motility (30). O-1602 also has pro-inflammatory and pro-atherogenic effects which are thought to be mediated by GPR55 (31).

An acute, single dose treatment of O-1602 in rodents increased food intake, via reduced expression of the anorexigenic neuropeptide cocaine- and amphetamine-regulated transcript (CART) (32). However, when lean rodents fed a standard chow diet were infused with O-1602 for 7 days, an increase in adiposity was observed despite no alterations to weight gain, food intake or individual fat pad mass (32).

Thus, as O-1602 and O-1918 may be able to mediate a number of physiological processes including the metabolic regulation of weight and appetite, it is hypothesized that these compounds will have an effect on obesity. Therefore, this study aimed to determine the effects that treatment with either O-1602 or O-1918 had in a diet-induced obese (DIO) rat model. Specifically, the objective of the study was to examine the effects of O-1602 and O-1918 on body weight, food consumption, body composition, organ weights, blood pressure and blood glucose control in a high-fat obesity model. Furthermore, this study aimed to elucidate whether these compounds elicited changes to signaling pathways in organs known to be affected by the obese state, including the kidneys and liver.

## Materials and methods

### Animals

All animal studies were conducted in accordance with the National Institutes of Health’s Guide for the Care and Use of Laboratory Animals. All animal-experimental procedures were approved by The Florey Institute of Neuroscience and Mental Health Animal Ethics Committee (AEC 11-036 and AEC 09-050) and performed at the Howard Florey Institute (Parkville, Victoria, Australia). Seven-week-old male Sprague–Dawley rats were sourced from the Animal Resource Centre (Canning Vale, Western Australia). Sprague–Dawley rats were selected due to their ability to gain weight on a high-fat diet (HFD). This strain of rat also shows a diverse response in weight gain following consumption of a HFD with some Sprague–Dawley rats being obese resistant and some being predisposed to obesity. Following acclimatization to experimental conditions the rats (weight 322.0 g ± 31.7, prior to commencing the HFD) were individually housed in a plastic tube with a secure stainless steel lid (dimensions width 27.5 × length 41 × height 25.5 cm) (R.E. Walters, Sunshine, Melbourne, Victoria, Australia) in an environmentally controlled laboratory (ambient temperature 22–24°C) with a 12 h light/darkness cycle (07:00–19:00).

### High-fat feeding and chronic treatment with pharmacological compounds O-1602 or O-1918 in DIO model

A cohort of rats (*n* = 8 per group) were randomly assigned to either the HFD (containing 21% crude fat, 40% digestible energy from lipids; sourced from Specialty Feeds Ltd., Glen Forrest, Australia; Table 1) or a chow diet (containing 10% digestible energy from lipids; sourced from Barastoc Ltd., Melbourne, Victoria, Australia; Table 1) (33) for 10 weeks. The list of ingredients for the SCD and HFD are outlined in Table 1 and comparison nutritional composition of the SCD and HFD is shown in Table 2. In a separate cohort, rats were fed a HFD (33) for 9 weeks. In this rat model, we have previously established that 9 weeks of DIO is sufficient to induce significant increases in body weight, body fat composition and blood pressure compared to standard chow lean controls (33). As the rats had a diverse response to the HFD, a group allocation was completed which matched animals according to weight, body composition, blood pressure, glucose tolerance and insulin sensitivity and the rats were then allocated to either DIO control (*n* = 11), DIO O-1602 (*n* = 6), or DIO O-1918 (*n* = 9) groups. O-1918 and O-1602 were sourced from Tocris Bioscience (Bristol, UK). Rats consuming a HFD, for a further 6 weeks were maintained on the HFD and treated daily with either a vehicle 0.9% isotonic saline solution containing 0.75% Tween 80 (DIO + control), 5 mg/kg of O-1602 (DIO + O-1602) or 1 mg/kg of O-1918 (DIO + O-1918) with compounds dissolved in the vehicle solution, and administered via intraperitoneal (ip.) injection. The dosages of O-1602 and O-1918 were chosen based on previous literature demonstrating that the compound reduces colitis scores in a colitis model (34), and inhibits the hypotensive effects of Abn-CBD in mice (23), respectively. Access to food and water was maintained, *ad libitum,* throughout the duration of the study.

**Table 1:** Ingredients listed in diets.

High-fat diet (SF00-219)	Standard chow diet
Casein (acid)	Cereal grains and cereal by-products
Sucrose	Legumes and legume by-products
Clarified butter (ghee)	Vegetable protein meals
Cellulose	Fats and oil
Wheat starch	Vitamins
DL Methionine	Minerals
Calcium carbonate	*Yucca schidigera* extracts
Sodium chloride	
AIN93 trace minerals	
Potassium citrate	
Potassium dihydrogen phosphate	
Potassium sulfate	
Choline chloride (75%)	
SF00-219 vitamins or AIN93 vitamins	
Cholesterol	
Oxicap E2

**Table 2:** Comparison of nutritional composition of diets.

Nutritional composition	High-fat diet (SF00-219)	Standard chow diet
Protein	19.0%	20%
Total fat	21.0%	5%
Crude fiber	4.7%	5%
Digestible energy	19.4 MJ/kg	17.25 MJ/kg
% Total calculated digestible energy from lipids	40.0%	10.7%
% Total calculated digestible energy from proteins	17.0%	19.7%

Following the conclusion of the pharmacological treatment period, rats were deeply anesthetized using 3% isoflurane (Abbott), skeletal muscles were extracted and then the rats were administered an ip. injection of sodium pentabarbitone (100 mg/kg; Virbac, Peakhurst, Australia) and killed via cardiac puncture. While the DIO 10 week group of rats were administered an ip. injection of sodium pentabarbitone and then killed via cardiac puncture. Organs including the heart, kidney, liver and adipose tissue fat pads were then removed postmortem, weighed individually and then the tissues were stored at −80°C for further analysis.

### Physiological measurements

Body weight and food consumption of the rats were recorded daily throughout the experimental treatment period. Daily food consumption was determined by collecting and weighing food pellets remaining in the cage each day and ensuring that any visible spillage within the cage was also collected and weighed. Total body composition was measured at baseline (after 9 weeks of DIO), 3 weeks into treatment (after 12 weeks of DIO) and 6 weeks into treatment (at the 15 week conclusion of DIO) using an EchoMRI Whole Body Composition Analyzer (EchoMRI-900; EchoMRI, Houston, TX, USA) (33). Systolic and diastolic blood pressure measurements were obtained from conscious rats using a noninvasive tail-cuff method with volume pressure recording software CODA 2 (Kent Scientiﬁc, Torrington, CT, USA) (33) at baseline (after 9 weeks of DIO) and 6 weeks into treatment (at the 15 week conclusion of DIO). Glucose tolerance (2 g/kg glucose) and insulin sensitivity (0.75 U/kg insulin) tests were measured at baseline (after 8 weeks of DIO) and toward the end of the pharmacological treatment period 5 weeks into treatment (after 14 weeks of DIO). The glucose or insulin bolus used for the glucose tolerance test and insulin sensitivity test were administered using an ip. injection. Rats were fasted overnight for the glucose tolerance test and for two hours prior to commencing the insulin sensitivity test. Blood glucose in response to glucose and insulin was analyzed as delta area under the curve (delta AUC) using GraphPad Prism Software (33). To assess insulin sensitivity further the rate of glucose utilization K_ITT_ (%/m) = [(ln1 − ln2)/(t2 − t1)] × 100 (35, 36), the half-life of glucose t½ (min) = (0.693/K_ITT_) × 100 (36) and maximal decline of glucose (mmol/L) were also calculated.

### Hydroxyproline analysis

To determine total collagen content and concentration from the heart and liver of rats subjected to DIO and treated with or without O-1602, a colorimetric-based hydroxyproline assay was utilized to measure fibrosis as previously described (37).

### Renal measurements

Urine was collected over a 24 h period using a metabolic cage. Measurements of urinary albumin (ALPCO Diagnostics, Salem, NH, USA) and both urinary and plasma creatinine (Cayman Chemical Company, Ann Arbor, MI, USA) were determined using commercially available kits, according to the manufacturer’s instructions as previously described (33) and were evaluated at baseline and during the final week of the pharmacological intervention (pre- and posttreatment periods). Estimated creatinine clearance was measured once and was determined using blood collected at the experimental end point, and the final 24 h urine collection. Estimated creatinine clearance was determined using the formula (Urinary vol (mL/min) × urinary creatinine concentration (mg/dL))/plasma creatinine (mg/dL) and was adjusted for body weight. Histological analysis of the glomerular area and tubular cross-sectional diameter was imaged at 200× magniﬁcation (Carl Zeiss). Glomerular area and tubular diameter was measured using image analysis software (Axiovision 4.8; Carl Zeiss) (33).

### Plasma analysis

Following cardiac puncture, blood was transferred into 10 mL Ethylenediaminetetraacetic acid (EDTA) BD Vacutainer tubes (McFarlene Medical, Surry Hills, Victoria, Australia) and kept on ice until samples were centrifuged at 4000 × ***g*** for 10 min at 4°C. The plasma layer was aspirated and stored until further analysis at −80°C. Plasma concentrations of transforming growth factor beta 1 (TGF-β1) (Promega), adiponectin (AdipoGen, Liestal, Switzerland), glucagon-like peptide 1 (GLP-1), ghrelin, leptin (Bioplex hormone immunoassay; Bio-Rad Laboratories), erythropoietin (EPO), growth regulated α protein/keratinocyte chemoattractant (GROC/KC), interferon gamma (IFN-γ), interleukin 1 alpha (IL-1α), interleukin 1 beta (IL-1β), monocyte chemotactic protein 1 (MCP-1), interleukin 2 (IL-2), interleukin 4 (IL-4), interleukin 5 (IL-5), interleukin 6 (IL-6), interleukin 10 (IL-10), interleukin 17 alpha (IL-17α), interleukin 18 (IL-18), macrophage colony stimulating factor (MC-SF), macrophage inflammatory protein 3 alpha (MIP-3α), regulated upon activation of normal T-cells expressed and secreted (RANTES), tumor necrosis factor alpha (TNFα), interleukin 12p70 (IL-12p70), vascular endothelial growth factor (VEGF) and interleukin 13 (IL-13) were analyzed in accordance with manufacturer’s instructions (Bioplex cytokine assay; Bio-Rad Laboratories). A number of samples within each group were out of range for some of the analytes tested, these include the following: plasminogen activator inhibitor 1 (PAI-1), granulocyte colony stimulating factor (G-CSF), granulocyte macrophage colony stimulating factor (GM-CSF) and interleukin 7 (IL-7). Plasma concentrations of aspartate aminotransferase (AST), alanine transaminase (ALT) and albumin (Randox Laboratories Ltd, Crumlin, County Antrem, UK) were analyzed at the University of Melbourne Veterinary Hospital (Werribee, Victoria, Australia) in accordance with manufacturer’s instructions.

### Western blotting

Protein was isolated as described previously (38, 39). Aliquots (40–100 μg) of the protein lysates were separated on a 7.5–20% SDS-PAGE gel and transferred to a nitrocellulose membrane. GPR55 (Novus Biologicals, Littleton, CO, USA), TGF-β1 (Abcam), collagen IV (Abcam), VEGF (Abcam) and β-actin (Sigma Aldrich) were detected using Western blot analysis from kidney lysate using specific antibodies. Secondary anti-mouse and anti-rabbit antibodies were purchased from Sigma Aldrich. Band densitometry was analyzed using Image Lab software (Bio-Rad Laboratories).

### Statistical analysis

The statistical package GraphPad 7.00 Prism Software was used to generate graphs and to perform all statistical analysis. All data are presented as mean ± s.e.m. Normality of data sets were determined using Sharipo–Wilk normality test. Differences between treatment groups were individually analyzed and compared to the DIO control group using either an independent *t*-test or Mann–Whitney U test for two group direct analyses. Alternatively a two-way ANOVA and a Bonferroni’s multiple comparisons test was used for measurements that contain more than one time point. Significance was accepted when *P* < 0.05.

## Results

### Physiological effects

**Figure 1:**
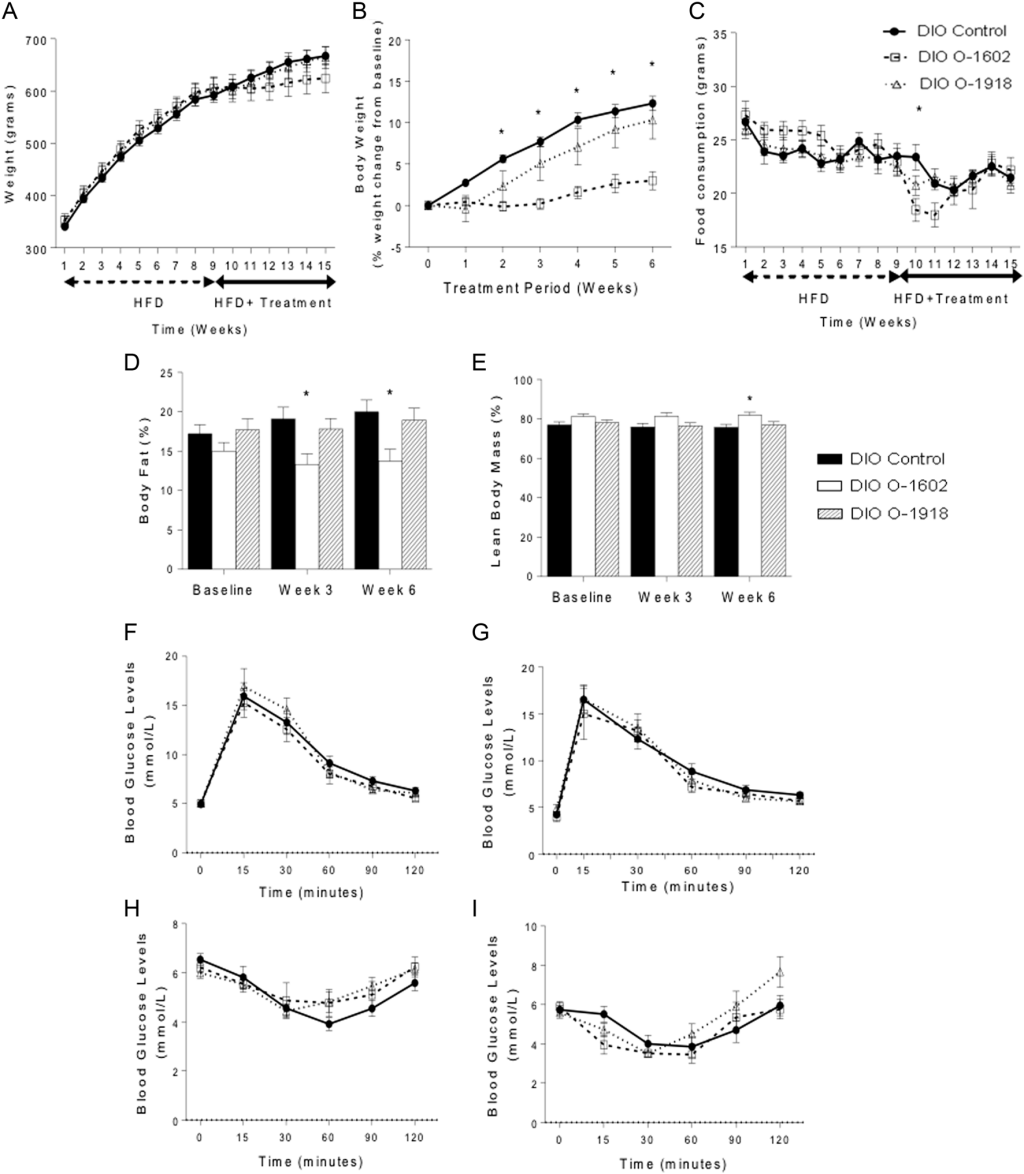
Physiological outcomes obtained from rats fed a high-fat diet for 9 weeks to induce obesity, then the diet-induced obese (DIO) rats were allocated to one of three treatment groups based on their characteristics including weight, body composition, blood pressure, glucose tolerance and insulin sensitivity and then treated with either a vehicle (DIO control) (*n* = 11), O-1602 (DIO O-1602) (*n* = 6) or O-1918 (DIO O-1918) (*n* = 9) for a period of 6 weeks. (A) Body weight (grams) for both DIO and the treatment period. (B) Weight change (% from baseline DIO) over the treatment period. (C) Food consumption (grams) for both DIO and the treatment period. (D) Body fat % over the treatment period and (E) lean body mass % over the treatment period. (F) ip. GTT blood glucose levels (mmol/L) at week 8 prior to treatment. (G) ip. GTT blood glucose levels (mmol/L) at week 14 following treatment. (H) ip. IST blood glucose levels (mmol/L) at week 8 prior to treatment. (I) ip. IST blood glucose levels (mmol/L) at week 14 following treatment. Data was analyzed using a two tailed *t*-test to compare the DIO + control group to either the DIO + O-1602 group (*significance *P* < 0.05) or the DIO + O-1918 group (#significance *P* < 0.05). Data is represented as group average ± s.e.m.

In our DIO rat model, we had previously reported that rats fed a HFD for 9 weeks had a significantly greater body weight and body fat percentage when compared with rats fed a chow diet (33), indicating that the rats utilized in these experiments were all DIO prior to treatment. The body weight (grams) for both pharmacological treatment groups were not altered when compared with the DIO control group (Fig. 1A), however the body weight of the DIO + O-1602-treated rats (% weight change from baseline of DIO) was reduced from weeks 2 to 6 of the treatment period, while the body weight (% change from baseline of DIO) of the DIO + O-1918-treated rats was not significantly altered (Fig. 1B). In terms of body composition, the body fat percentage was reduced at weeks 3 and 6 and lean tissue mass was increased at week 6 for the DIO + O-1602-treated rats, but not significantly altered for the DIO + O-1918-treated group over the entire treatment period (Fig. 1D and E). Food intake was transiently reduced in the first week of treatment for the DIO + O-1602 group only (Fig. 1C). Blood pressure was not affected by treatment (Table 3). Glucose tolerance and insulin sensitivity all remained unchanged across all the treatment groups when compared to DIO + control (Fig. 1F, G, H and I and Table 3).

**Table 3:** The effect that chronic administration of either O-1602 or O-1918 have on physiological outcomes (pre- and posttreatment) in a diet-induced obese rat model.

Measurement	Treatment groups					
	DIO control		DIO O-1602		DIO O-1918	
	Pretreatment	Posttreatment	Pretreatment	Posttreatment	Pretreatment	Posttreatment
Weight (g)	592 ± 13	667 ± 17	605 ± 22	624 ± 27	604 ± 21	664 ± 21
Food consumption (g)	23.5 ± 1.1	21.7 ± 0.5	23.0 ± 1.2	22.2 ± 1.2	22.5 ± 0.9	20.8 ± 0.8
Total body fat mass (%)	17.2 ± 1.2	20.0 ± 1.6	15.0 ± 1.1	13.8 ± 1.5*	17.7 ± 1.4	19.0 ± 1.5
Total lean body mass (%)	77.1 ± 1.5	75.8 ± 1.5	81.3 ± 1.2	81.9 ± 1.6*	78.1 ± 1.4	77.1 ± 1.6
Diastolic blood pressure (mmHg)	105 ± 6 (*n* = 10)	105 ± 5	116 ± 72	114 ± 8	94 ± 4.3 (*n* = 8)	95 ± 3 (*n* = 6)
Systolic blood pressure (mmHg)	141 ± 4 (*n* = 10)	144 ± 5	159 ± 8	157 ± 9	133 ± 4 (*n* = 8)	140 ± 1 (*n* = 6)
Glucose tolerance (delta AUC)	565 ± 73	613 ± 85	473 ± 88	600 ± 70	542 ± 67 (*n* = 8)	504 ± 58
Insulin sensitivity (delta AUC)	212 ± 31	205 ± 43	130 ± 39	198 ± 34	126 ± 29	128 ± 26
KITT (%/m)	0.9 ± 0.1	0.8 ± 0.2	0.8 ± 0.1	1.0 ± 0.2	0.6 ± 0.1	0.7 ± 0.1
t½ (min)	93 ± 14	149 ± 54	94 ± 13	100 ± 28	143 ± 36	116.6 ± 17.4
Maximal decline of glucose	3.7 ± 0.3	3.5 ± 0.5	4.3 ± 0.6	2.9 ± 0.3	4.3 ± 0.2	3.4 ± 0.2

Physiological outcomes were obtained from rats fed a high-fat diet for 9 weeks to induce obesity, then the diet-induced obese (DIO) rats were treated with either a vehicle (DIO control) (*n* = 11), O-1602 (DIO O-1602) (*n* = 6) or O-1918 (DIO O-1918) (*n* = 9) for a period of 6 weeks. Where data points were missing the specific *n* value is included on table next to the mean value for that measurement and group. After determining the normality of each data set, data was analyzed using either a two tailed *t*-test or Mann–Whitney U test to compare the DIO + control group to either the DIO + O-1602 group or the DIO + O-1918 group (*significance *P* < 0.05). Data is represented as average ± s.e.m. Bold indicates statistical significance.

### Effects on organ weights

The DIO + O-1602-treated group had reduced epididymal fat depots when compared with the DIO control group, there was also a nonsignificant trend (*P* = 0.077, total mass and *P* = 0.074, percentage of body weight) for peri-renal fat pad depots to be reduced (Table 4). These findings support the observations of a reduction in body fat percentage, while brown adipose tissue (BAT) depots were not altered by treatment with this compound. There was also an increase in kidney and liver mass in the DIO + O-1602-treated group while heart mass was unaffected by the chronic treatment (Table 4).

**Table 4:** The effect that chronic administration of either O-1602 or O-1918 has on organ weights in a diet-induced obese rat model.

Measurement	Treatment groups		
	DIO control	DIO O-1602	DIO O-1918
Body weight at time of death (grams)	680 ± 20	632 ± 27	669 ± 20
Heart mass (grams)	1.7 ± 0.1 (*n* = 7)	1.7 ± 0.1	1.6 ± 0.1
Heart mass/body weight (%)	0.3 (*n* = 7)	0.3	0.2
Kidney mass (grams)	1.7 ± 0.1	**2.0** ± 0.1*	1.6 ± 0.1
Kidney mass/body weight (%)	0.3	0.3*	0.3
Epididymal fat pad mass (grams)	10.8 ± 0.8	**7.4** ± 0.7*	8.8 ± 0.8
Epididymal fat pad mass/body weight (%)	1.6 ± 0.1	**1.2** ± 0.1*	1.3 ± 0.1
Peri-renal fat pad mass (grams)	12.7 ± 1.3	9.1 ± 0.8	12.2 ± 1.3
Peri-renal fat pad mass/body weight (%)	1.8 ± 0.1	1.4 ± 0.1	1.8 ± 0.2
Brown fat pad mass (grams)	1.1 ± 0.1 (*n* = 10)	0.8 ± 0.1	**0.8** ± 0.1*
Brown fat pad mass/body weight (%)	0.2 (*n* = 10)	0.1	0.1*
Liver mass (grams)	23.1 ± 1.0	23.5 ± 0.9	23.2 ± 1.4
Liver mass/body weight (%)	3.4 ± 0.1	**3.7** ± 0.1*	3.5 ± 0.2

Organs were obtained from rats fed a high-fat diet for 9 weeks to induce obesity, then the diet-induced obese (DIO) rats were treated with either a vehicle (DIO control) (*n* = 11), O-1602 (DIO O-1602) (*n* = 6) or O-1918 (DIO O-1918) (*n* = 9) for a period of 6 weeks. Where organ weights for data sets were missing the specific *n* value is included on table next to the mean organ weight for that group. After determining the normality of each data set, data was analyzed using either a two tailed *t*-test or Mann–Whitney U test to compare the DIO + control group to either the DIO + O-1602 group or the DIO + O-1918 group (*significance *P* < 0.05). Data is represented as average ± s.e.m. Bold indicates statistical significance.

The DIO + O-1918-treated group had reduced BAT depots when compared with the DIO control group. Other tissues, including WAT epididymal and peri-renal fat pads, liver, kidney and heart mass, were not altered by chronic treatment with O-1918 in this DIO model (Table 4).

### Effects on circulating plasma hormones and cytokines

The plasma hormone analysis shows that DIO rats treated with O-1602 or O-1918 had reduced circulating leptin and ghrelin concentrations when compared to the DIO control group. Conversely other hormones including glucagon, GLP-1 and adiponectin plasma concentrations were not altered by either of the pharmacological treatments (Table 5).

**Table 5:** The effect that chronic administration of either O-1602 or O-1918 has on either circulating plasma hormones or cytokines in a diet-induced obese rat model.

Analyte	DIO Control	DIO O-1602	DIO O-1918
Hormones			
Glucagon (pg/mL)	345 ± 50	241 ± 42	291 ± 58
GLP-1 (pg/mL)	236± 76	119 ± 45	319 ± 99 (*n* = 8)
Ghrelin (ng/mL)	4 ± 0.4	**2** ± 0.2*	**2** ± 0.2*
Leptin (ng/mL)	10 ± 1	**3** ± 0.4*	**6** ± 1*
Adiponectin (µg/mL)	11 ± 0.8	12 ± 2	12 ± 1
Cytokines			
EPO (pg/mL)	826 ± 165	1263 ± 322	1675 ± 439
GRO/KC(pg/mL)	254 ± 76 (*n* = 10)	367 ± 68	213 ± 36
IFN-γ (pg/mL)	446 ± 177 (*n* = 8)	578 ± 163 (*n* = 5)	664 ± 184
IL-1α (pg/mL)	170 ± 48 (*n* = 10)	300 ± 88	**459** ± 129*
IL-1β (ng/mL)	6 ± 2	8 ± 3	10 ± 3
MCP-1 (ng/mL)	1 ± 0.2 (*n* = 9)	1 ± 0.1	1 ± 0.2
IL-2 (pg/mL)	341 ± 71	588 ± 149	**860** ± 201*
IL-4 (pg/mL)	230 ± 72	337 ± 86	456 ± 108
IL-5 (pg/mL)	373 ± 71	527 ± 124	521 ± 94
IL-6 (pg/mL)	276 ± 108 (*n* = 9)	427 ± 112 (*n* = 5)	606 ± 217
IL-10 (ng/mL)	1 ± 0.4	1 ± 0.3	2 ± 0.4
IL-17α (pg/mL)	114 ± 30	203 ± 53	**271** ± 48*
IL-18 (ng/mL)	4 ± 1	7 ± 3	**8.0** ± 1.8*
MC-SF (pg/mL)	504 ± 34	423 ± 29	449 ± 43
MIP-3α (pg/mL)	118 ± 29	140 ± 30	190 ± 33
RANTES (pg/mL)	297 ± 64	520 ± 126	**724** ± 78*
TNFα (pg/mL)	185 ± 60	204 ± 52	270 ± 61 (*n* = 8)
IL-12p70 (pg/mL)	276 ± 90	470 ± 166	521 ± 154 (*n* = 8)
VEGF (pg/mL)	67 ± 22	80 ± 27	103 ± 30
IL-13 (pg/ mL)	108 ± 34	140.4 ± 46	203 ± 51 (*n* = 8)
TGFβ (ng/mL)	18 ± 3 (*n* = 8)	17 ± 2	20 ± 2 (*n* = 7)

Blood/plasma was obtained from rats fed a high-fat diet for 9 weeks to induce obesity, then the diet-induced obese (DIO) rats were treated with either a vehicle (DIO control) (*n* = 11), O-1602 (DIO O-1602) (*n* = 6) or O-1918 (DIO O-1918) (*n* = 9) for a period of 6 weeks. When data sets were out of range the changed *n* value is included on table next to the mean concentration for that group. After determining the normality of each data set, data was analyzed using either a two tailed *t*-test or Mann–Whitney U test to compare the DIO + control group to either the DIO + O-1602 group or the DIO + O-1918 group (*significance *P* < 0.05). Data is represented as average ± s.e.m. Bold indicates statistical significance.

The plasma cytokine analysis shows DIO + O-1602-treated rats had a nonsignificant trend for RANTES to be increased (*P* = 0.0981), while all of the other cytokines were not altered when compared to the DIO + control group (Table 5). The plasma cytokine analysis also shows that DIO + O-1918-treated rats had increased plasma pro-inflammatory cytokines when compared to the DIO + control group, including IL-1α, IL-2, IL-17α, IL-18 and RANTES. There was also a nonsignificant trend for EPO to be increased (*P* = 0.056).

### Effects on renal structure and function

**Figure 2:**
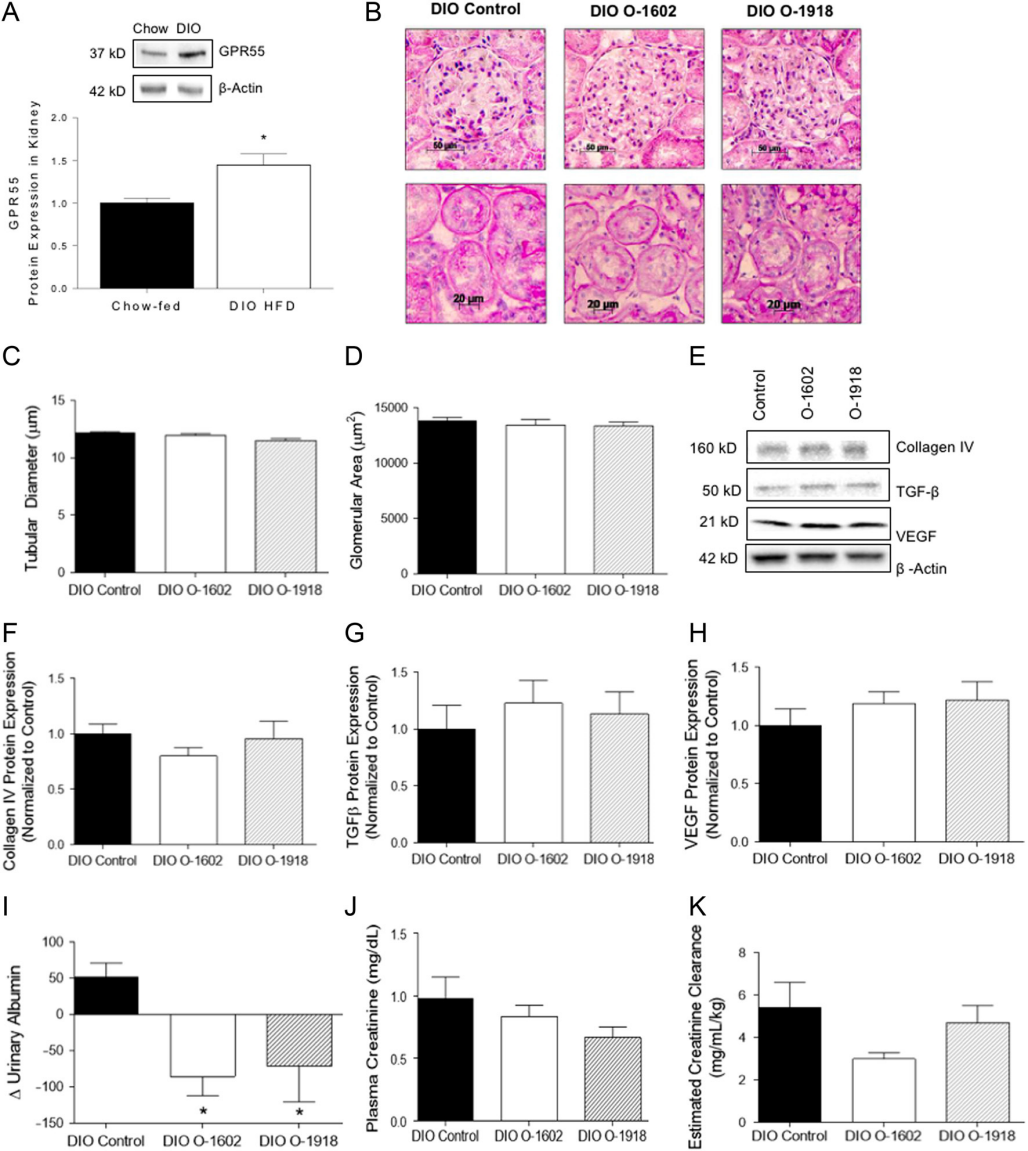
Renal structure and function obtained from rats fed a high-fat diet (HFD) for 9 weeks to induce obesity, then diet-induced obese (DIO) rats were treated with either a vehicle (DIO control) (*n* = 11), O-1602 (DIO O-1602) (*n* = 6) or O-1918 (DIO O-1918) (*n* = 9) for a period of 6 weeks or GPR55 expression in rats fed either a HFD (*n* = 8) or standard chow diet (chow) (*n* = 8) for 10 weeks. (A) Kidney protein expression of GPR55 increased in rats fed a HFD to induce obesity (DIO) (*n* = 8) compared to chow-fed for 10 weeks (*n* = 8). (B) H&E staining of glomerular and tubular cross-sectional areas shown at 200× magnification. (C) Quantification of tubular cross-sectional area (µm). (D) Quantification of glomerular cross-sectional area (µm^2^). (E) Western blots of fibrotic markers in kidney tissue. (F) Collagen IV expression in kidney tissue. (G) TGF-β expression in kidney. (H) VEGF expression in kidney. (I) Change in urinary albumin excretion (μg/mL). (J) Change in plasma creatinine concentration. (K) Estimated creatinine clearance, posttreatment (mg/mL/kg). Data was analyzed using a two tailed *t*-test to compare the DIO + control group to either the DIO + O-1602 group (*significance *P* < 0.05) or the DIO + O-1918 group (#significance *P* < 0.05). Data is represented as group average ± s.e.m.

We have shown for the first time that GPR55 is significantly increased in whole kidney tissue samples of DIO rats after 10 weeks of high-fat feeding (Fig. 2A). Histological analysis showed that treatment with O-1602 did not affect glomerular area or tubular cross-sectional diameter (Fig. 2B, C and D). Treatment with O-1918 did exhibit significantly reduced tubular cross-sectional area compared to DIO controls (Fig. 2B, C and D). No significant changes, however, were observed in the renal fibrotic markers collagen IV, TGF-β1 or VEGF protein in DIO rats treated with either O-1602 or O-1918 compared to DIO controls (Fig. 2E, F, G and H).

In DIO rats treated with either O-1602 or O-1918, urinary albumin excretion (Fig. 2I) was significantly reduced compared to DIO controls. No significant changes in plasma creatinine (Fig. 2J) or estimated creatinine clearance (Fig. 2K) were observed in DIO rats treated with either O-1602 or O-1918 compared to DIO controls.

### Effects on hepatic function

**Figure 3:**
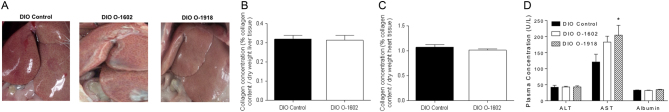
Liver or heart analysis obtained from rats fed a high-fat diet for 9 weeks to induce obesity, then the diet-induced obese (DIO) rats were treated with either a vehicle (DIO control) (*n* = 8), O-1602 (DIO O-1602) (*n* = 6) or O-1918 (DIO O-1918) (*n* = 9) for a period of 6 weeks. (A) Representative picture of DIO + control, DIO + O-1602 and DIO + O-1918-treated whole liver. (B) Liver collagen concentration. (C) Heart collagen concentration. (D) Plasma concentration of liver function analytes. Data was analyzed using a two tailed *t*-test to compare the DIO control group to either the DIO + O-1602 group (*significance *P* < 0.05) or the DIO + O-1918 group (#significance *P *< 0.05). Data is represented as group average ± s.e.m.

Livers from DIO + O-1602-treated rats had abnormal and dark appearing pigments when compared to the DIO + control group (Fig. 3A and B). As a result of these abnormal and dark appearing pigments and the need to euthanize one rat within the DIO + O-1602 treatment group (one rat was removed from all analysis leaving *n* = 6) as a result of ongoing diarrhea and rapid weight loss, no further animals were treated with O-1602. Despite these observations fibrotic marker hydroxyproline, which is a component of liver and cardiac collagen content in the DIO + O-1602-treated group, was not altered in either tissue (Fig. 3B and C). Plasma liver function test indicated a nonsignificant trend for AST to be increased (*P* = 0.078) in the DIO + O-1602-treated group when compared to controls, while circulating ALT and albumin were not altered. DIO + O-1918-treated rats had no visual difference in liver appearance when compared to the DIO + control group (Fig. 3A and C). Circulating plasma concentration of AST was increased while ALT and albumin were not altered.

## Discussion

Our study is the first to investigate O-1602 or O-1918 and their effects on whole body energy homeostasis, renal and hepatic function in a DIO rat model. For the first time we showed in DIO rats that GPR55 expression was elevated in renal tissue. Research focusing on GPR55 in obesity shows that GPR55 expression is significantly greater in the adipose tissue of obese humans when compared with lean individuals (40), these data are consistent with our observations of GPR55 expression in the DIO rat kidney compared to chow-fed controls. Recently, GPR55 has been shown to be expressed in skeletal muscle of rat and human, the same research group concluded that GPR55 is a positive regulator of insulin action and adipogenesis (41). These finding were consistent with Meadows *et al*. (57) that previously had found that GPR55 knockout mice have increased insulin resistance and adiposity as a result of reduced physical activity but had not confirmed expression in the skeletal muscle. Further, rats fed a low fat diet have greater GPR55 expression in white adipose tissue when compared with HFD fed rats, although the duration in which the diets were consumed is unclear. Therefore GPR55 expression appears to vary depending on species, dietary intake and tissue type, and may also be a beneficial therapeutic target for obesity-related comorbidities such as type 2 diabetes mellitus.

Further, O-1602 treatment in this DIO model had significant effects on several metabolic parameters, including reduced weight gain (% weight change from baseline) and body fat %, increased lean mass and a transient reduction in food intake. Our findings clearly indicate that O-1602 has a role in metabolic homeostasis. Previous research in a lean Sprague–Dawley model administered O-1602 at a lower dosage and for a shorter timeframe than our study (0.1–1 mg/kg for 1 week) found no reduction in food intake or body weight and increased body fat mass (32). Further, an acute dose of O-1602 (200 μg/kg, IP) administered to GPR55 knockout mice increased food intake (32) suggesting this compound may elicit effects on receptor(s) other than GPR55 such as GPR18 (21, 42). In addition to the reduced body fat observed in the DIO + O-1602-treated rats, the reduced body weight could also be reflective of alterations in the functioning of various other tissues due to the expression profiling of receptors targeted by O-1602, including GPR55 which has been demonstrated to alter transit time and expressed in the myenteric neurons in the colon (43, 44).

In contrast to the O-1602 treatment, O-1918 treatment in DIO rats did not alter a range of metabolic measures, but did reduce BAT mass when compared to the DIO + control group. Two hormones involved in regulating food intake; leptin and ghrelin were both decreased in response to treatment with O-1602 and O-1918. Leptin and ghrelin have opposing roles on food intake and appetite control; leptin induces satiety, while ghrelin increases appetite (45). Obesity is accompanied by a dysregulation in leptin signaling where hyperleptinemia (46) and leptin resistance (47) are evident. We have previously shown in this model of obesity that DIO + control treated rats had increased circulating plasma leptin concentrations compared to chow-fed control rats (48). Both leptin and ghrelin concentrations are influenced by food intake (49, 50) and the release of ghrelin is produced during the fast state and inhibited during the fed state (50), whereas leptin secretion is promoted during food intake and in the fed state and is decreased during starvation (49). Plasma leptin concentrations reflect total adipose tissue stores (51). The DIO + O-1602-treated rats had reduced epididymal fat pad mass and total body fat percentage which reflects total adipose tissue store, this potentially explains the decreased circulating leptin concentrations observed in the DIO + O-1602 group of rats. A relationship between cannabinoids and leptin exists (52), with mRNA expression of GPR55 increasing following leptin administration in starved rodents, reverting the GPR55 mRNA expression levels back to the same concentrations as rats with *ad libitum* access to chow-fed (53). The interaction between leptin and GPR55 could influence leptin plasma concentrations with O-1918 treatment. Despite the alterations in leptin and ghrelin, no alterations in GLP-1, glucagon and adiponectin were observed.

This study is first to look at the effect of O-1602 and O-1918 on several circulating cytokines and chemokines in DIO. Cytokines and chemokines were not significantly altered following O-1602 treatment. However, O-1918 treatment in DIO increased pro-inflammatory cytokines including IL-1α, IL-2, IL-17α, IL-18 and RANTES. The DIO + O-1918-treated rats had reduced BAT depots compared with the DIO control rats. In DIO rodents, blocking CB_1_ increases temperature of BAT and upregulates UCP-1 (54). In this study, we measured BAT deposits but changes in function of this tissue with the treatment of O-1918 were not investigated. Increased pro-inflammatory cytokines (associated with the development of insulin resistance), accompanied by the decrease in BAT could be interrelated. Over-activity of pro-inflammatory cytokines can lead to dysfunction and apoptosis in brown adipocytes in a murine cell culture model mediated by UCP-1 and β-Klotho suppression (55). Rebiger *et al*. (55) investigated pro-inflammatory cytokines IL-1β, TNFα and IFN-γ, which contrasts with our results as these cytokines were not altered. However, as administration of O-1918 in DIO increased several other pro-inflammatory cytokines these cytokines may have contributed to apoptosis in the BAT; however, further investigation is required to elucidate this. O-1918 in DIO did not alter glucose tolerance or insulin sensitivity in our study; however, the rats were not glucose intolerant or insulin resistant as a result of the HFD used (33), which is in line with previous research (56). Therefore, the effect that O-1918 had on impaired glucose homeostasis could not be assessed in this model; however, our plasma results suggest that O-1918 does not impair glucose tolerance or insulin sensitivity. GPR55 knockout mice have increased adiposity and insulin resistance (57), given that O-1918 is a putative antagonist for GPR55 and that obesity and insulin resistance are associated with chronic low-grade inflammation (58, 59), this could account for the increased circulating pro-inflammatory cytokines observed in this study.

O-1602 treatment significantly reduced urinary albumin, in conjunction with abrogation of weight gain. Plasma creatinine, creatinine clearance or cytokine profile in renal tissue were not altered in DIO + O-1602 or O-1918-treated rats. The reduced weight gain observed in our model of DIO + O-1602-treated rats potentially mediated the significant improvements in kidney function, including reduced urinary albumin. Histological examination showed no signs of structural alterations to the kidney induced by treatment with O-1602 compared to DIO + controls and the renal cytokine profile was also not altered. Both gross kidney weight and kidney weight as a percentage of total body weight were significantly higher in O-1602+DIO rats. Improved albuminuria in O-1602-treated rats may be independent of structural changes to the kidney (particularly as no changes to tubular diameter or glomerular area was detected in DIO + O-1602-treated rats), as male Wistar rats infused with leptin showed increased proteinuria and albuminuria (60). Significant reduction in weight gain exhibited by DIO + O-1602-treated rats occurred concurrently with reduced circulating plasma leptin concentrations. Although the mechanism of leptin-induced proteinuria has yet to be elucidated, it is postulated that changes to proteinuria in leptin-infused animals may be caused by increased renal expression of TGF-β1 (60, 61). As DIO + O-1602-treated rats in our study showed no changes to renal cytokine profile, including renal expression of TGF-β1, it is unlikely that O-1602 mediates improved albuminuria via this pathway.

This study demonstrated that treatment with O-1918 leads to significant reductions in urinary albumin excretion and reduced renal tubular diameter compared to DIO + controls. These improvements occurred in the absence of reduced weight gain; however, as with the O-1602 treatment, circulating plasma leptin was significantly reduced compared to DIO + controls. This occurred in conjunction with no significant changes to gross kidney weight; however, a significant reduction in renal tubular diameter was observed. Previously, we identified that renal tubular cross-sectional area is significantly greater in rats with DIO after 10 weeks compared to lean chow-fed rats (48). Alterations to renal tubular architecture by treatment with O-1918 may contribute to reduced albumin urinary excretion in rats with DIO. Studies examining renal hypertrophy under pathophysiological conditions focus on protein synthesis; however, increased expression of collagen, TGF-β1, collagen IV and VEGF expression were not altered in this study. Hypertrophy may also emanate from a decrease in protein catabolism (62); however, exploring the process of protein breakdown in tubular cells was beyond the scope of this study but may be a mechanism by which O-1918 regulates renal hypertrophy. Other measures of renal function including plasma creatinine and estimated creatinine clearance were not altered with either treatment.

In DIO + O-1602-treated rats, the hepatic morphology observed suggests that this compound elicits undesirable effects, while DIO + O-1918 treated rats had similar liver morphology to DIO + controls. Currently, no studies have investigated the effects of O-1602 in the liver in DIO and therefore further investigation is required. Our data indicates that O-1602 causes an enlarged liver with abnormal dark pigmentation, and a nonsignificant trend for increased plasma AST, while other markers of liver function (ALT, albumin) were not altered. AST and ALT are both markers of hepatocyte integrity (63), while AST is also found in non-hepatic tissues, it is highly concentrated in the liver (63). Since the fibrotic marker hydroxyproline in both the liver and heart tissue was not altered in the DIO + O-1602-treated rats, this suggests that O-1602 is not causing fibrosis. Therefore, further investigation into the causes of the changes in liver morphology is required.

In conclusion, this study has for the first time focused on the role of atypical cannabinoid ligands O-1602 and O-1918 as therapeutics in obesity. These compounds attenuate some aspects of metabolic dysfunction associated with obesity. While O-1602 had beneficial effects on whole body composition and reduced albuminuria in obesity, the compound appeared to have adverse side effects particularly in the liver suggesting O-1602 may not be a suitable compound as an anti-obesity pharmaceutical at the dosage utilized in this study. Future research should focus on the G Protein-Coupled Receptors that O-1602 targets (such as GPR55 and GPR18) as modulating these receptors using different ligands may be of benefit when targeting specific tissues such as the adipose tissue or the kidney to help overcome the adverse effects observed with O-1602 treatment in this study. While O-1918 reduced BAT mass it did not alter total body fat or lean tissue mass, the compound did however help reduce albuminuria and reduced renal tubular hypertrophy. This study shows for the first time that O-1918 may be beneficial for ameliorating the renal structural and functional damage which occurs in response to a HFD. Therefore O-1918 maybe a beneficial compound in the treatment of obesity-related kidney disease. Further investigation into the mechanisms of action of O-1602 and O-1918 in the DIO state is required.

## Declaration of interest

The authors declare that there is no conflict of interest that could be perceived as prejudicing the impartiality of the research reported.

## Funding

This work was supported by the Allen Foundation (D H H, A J M), and through the Australian Government’s Collaborative Research Networks (CRN) program (A J M). Scholarship funding by Australian Postgraduate Award (K A J, L O) and Australian Rotary Health and the Rotary Club of Ballarat South (A C S); and Fellowship funding (C S S) by the National Health & Medical Research Council (NHMRC) of Australia [GNT1041766].

## Author contribution statement

A C S, K A J, L O, M L M, A J M and D H H participated in research design. A C S, K A J, L O and C S S conducted the experiments. All authors listed were involved in data analysis and contributed to writing and editing the manuscript.
